# A translational approach to capture gait signatures of neurological disorders in mice and humans

**DOI:** 10.1038/s41598-017-03336-1

**Published:** 2017-06-12

**Authors:** Lauren Broom, Brian A. Ellison, Audrey Worley, Lara Wagenaar, Elina Sörberg, Christine Ashton, David A. Bennett, Aron S. Buchman, Clifford B. Saper, Ludy C. Shih, Jeffrey M. Hausdorff, Veronique G. VanderHorst

**Affiliations:** 1 Department of Neurology, Division of Movement Disorders, Beth Israel Deaconess Medical Center, Harvard Medical School, Boston, MA 02215 USA; 2 Rush Alzheimer’s Disease Center, Rush University Medical Center, Chicago, Il 60612 USA; 3 Center for the Study of Movement Cognition and Mobility, Tel-Aviv Sourasky Medical Center, Tel Aviv, 64239 Israel; 4 Sagol School of Neuroscience and Sackler Faculty of Medicine, Tel Aviv University, Tel Aviv, 69978 Israel

## Abstract

A method for capturing gait signatures in neurological conditions that allows comparison of human gait with animal models would be of great value in translational research. However, the velocity dependence of gait parameters and differences between quadruped and biped gait have made this comparison challenging. Here we present an approach that accounts for changes in velocity during walking and allows for translation across species. In mice, we represented spatial and temporal gait parameters as a function of velocity and established regression models that reproducibly capture the signatures of these relationships during walking. In experimental parkinsonism models, regression curves representing these relationships shifted from baseline, implicating changes in gait signatures, but with marked differences between models. Gait parameters in healthy human subjects followed similar strict velocity dependent relationships which were altered in Parkinson’s patients in ways that resemble some but not all mouse models. This novel approach is suitable to quantify qualitative walking abnormalities related to CNS circuit dysfunction across species, identify appropriate animal models, and it provides important translational opportunities.

## Introduction

Walking is a complex behavior that requires not only control of initiation and termination of locomotion, but also ongoing adjustments of speed, stride length, cadence, direction, and posture in response to dynamic internal and external cues. Walking can be affected in many different ways secondary to musculoskeletal or cardiovascular problems, as well as dysfunction of the peripheral or the central nervous system (CNS). Gait disorders due to specific CNS circuit pathologies can be clinically recognized based upon phenomenology, such as shuffling gait or festination in Parkinson’s disease or ataxic gait in cerebellar disorders ^1, 2^. The wide variety of causal conditions and subtypes of gait disorders illustrate that abnormal function or pathology in multiple CNS networks differentially affect gait.

Human gait can be objectively measured using direct or indirect kinematic, spatial or temporal measures via video-analysis, footfall studies, or wearable devices. Advances in imaging technology have allowed visualization of putative supratentorial circuitries involved in gait control^3–9^. However, the functional-anatomical basis for the various gait abnormalities so readily recognized in clinical settings remains poorly understood as approaches to isolate relevant networks during life are relatively limited. This gap in knowledge stands in the way of the development of specific, circuit based treatment strategies targeted to the various specific gait abnormalities.

Animal models offer a means to circumvent current barriers of studying gait in humans. In mice, highly selective approaches are now available to identify components of CNS circuits from cortex to spinal cord that mediate or modulate locomotion, including walking^10–13^. Effects of interventions can then be measured using various types of gait analysis^14, 15^ yielding measures similar to those obtained in human gait studies.

However, there are several factors that complicate analysis of gait metrics both in human and animal research. Firstly, many gait parameters change as a function of velocity even under “normal” conditions and it has been challenging to factor in these velocity-related changes while staying true to the biological properties of the data in animal models^16–20^ as well as in human subjects^21–23^. As a consequence, analysis is often simplified leading to loss of data. In addition, the presumed incompatibility of quadruped animal models and biped human gait remains a major barrier for translation.

In this study, we focus on similarities, rather than differences, in the velocity dependent relationships of a subset of human and mouse gait parameters. We take advantage of the relationships of each of the gait parameters as a function of velocity by establishing regression models that reproducibly capture these relationships or gait signatures during walking in mouse cohorts under baseline conditions. Following validation, we assessed whether gait signatures are perturbed following induction of parkinsonism in 3 different mouse models, with each cohort serving as its own control. Finally, we visualized data sets of human control subjects and patients with Parkinson’s disease (PD) in a similar velocity-dependent way. Our results demonstrate a rigorous mathematical analysis model of walking that can allow comparison of animal models of gait disorders with human disease. This opens the way for translational approaches to study the circuitry that underlies those abnormal gaits and find ways to correct them.

## Results

### Graphical summaries of gait metrics reveal their velocity dependent nature

**Figure 1:**
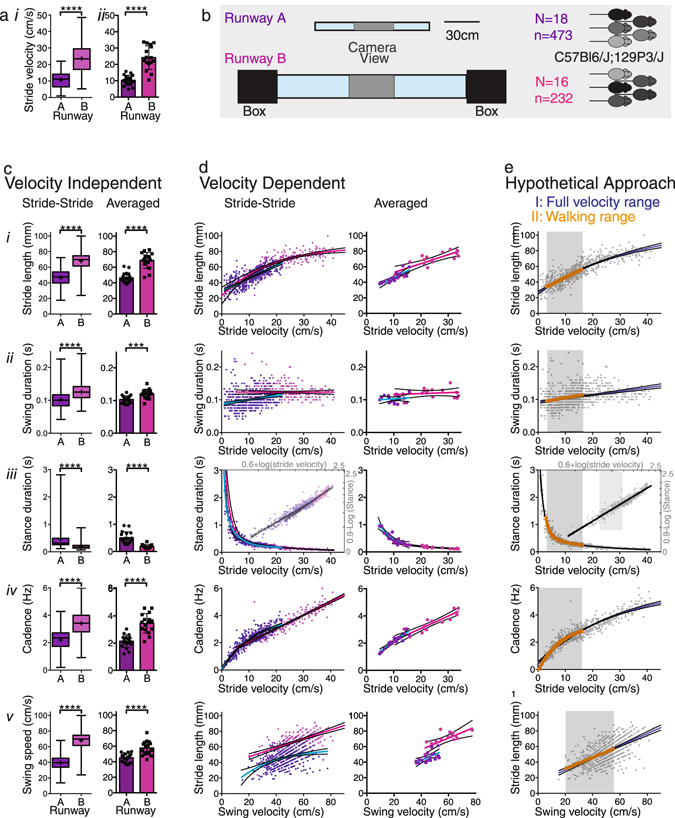
Visualization of spatial and temporal gait parameters. (**a**) Box and whisker plots (*i*) showing the distribution and comparisons of stride-to-stride velocity data collected on Runways A and B (Mann Whitney Rank test; center representing median, center cross representing mean, bars representing minimum and maximum), and (*ii*) bar graphs with scatter plots of average stride velocity (2-tailed t-test; error bars indicate standard deviation). (**b**) Schematic representation of the dimensions of runways A and B. Video recordings covered the middle portion (gray shaded area). (**c**) Stride length (*i*), swing duration (*ii*), stance duration (*iii*), cadence (*iv*) and swing speed (*iv*) represented as stride-to-stride data in box and whisker plots and as averaged data in bar graphs with scatter plots. See Supplementary Table S3 for statistical test results. (**d**) Visualization of gait parameters plotted as a function of stride velocity. Each of the parameters behaves differently as a function of velocity, as illustrated by best fit non-linear regression curves (colored lines) and 95% confidence bands (black lines). Panel iii contains a log transformation of the stance data (opaque). (**e**) Example of how regression models capture stride-to-stride data of the full velocity range versus walking range (gray panels).

We obtained spatial (stride length) and temporal gait measurements (stance and swing duration, cadence, swing speed) that are known to be speed dependent from high speed video recordings at the middle portion of 2 differently sized runways (Fig. 1; datasets 1 and 2 consisting of C57Bl6/J; 129P3/J mice; Supplementary Table S1). Mice moved at significantly higher speed (Fig. 1a) on the larger runway, designed to fit mice and rats, compared to a runway designed to fit mice (Fig. 1b). In these matched cohorts, spatial and temporal gait parameters obtained on the 2 runways differed markedly (Fig. 1c), with stride length, swing duration, cadence and swing velocity being larger or longer and swing duration being shorter on the larger runway. These differences were also observed in pure C57Bl6J mice (dataset 3, Supplementary Table 1; data not shown). These differences are due to the unique velocity dependent relationships of each of the gait parameters, which only become visible when plotting the stride-to-stride data for each of the gait parameters as a function of gait velocity (Fig. 1d). These relationships differ for each of the gait parameters examined and behave differently for slower versus faster ranges of velocity, i.e. the relationships are not linear throughout the entire velocity range.

These characteristics pose a challenge when analyzing gait characteristics during ground walking, as variable speeds occur from trial to trial and the preferred velocity often changes from baseline following experimental interventions. Representation of gait parameters irrespective of velocity, whether stride-to-stride or as averaged data sets (Fig. 1c), does not take these relationships into account and will only be valid if velocity distributions between groups are the same. For the same reason, averaged parameters plotted as a function of velocity have wide confidence intervals (Fig. 1d). Altogether, visualization of stride-to-stride data as a function of velocity provides insights into the characteristics of spatial and temporal gait parameters.

### Distinguishing changes in gait metrics due to a change in velocity versus a change in gait signature

**Figure 2:**
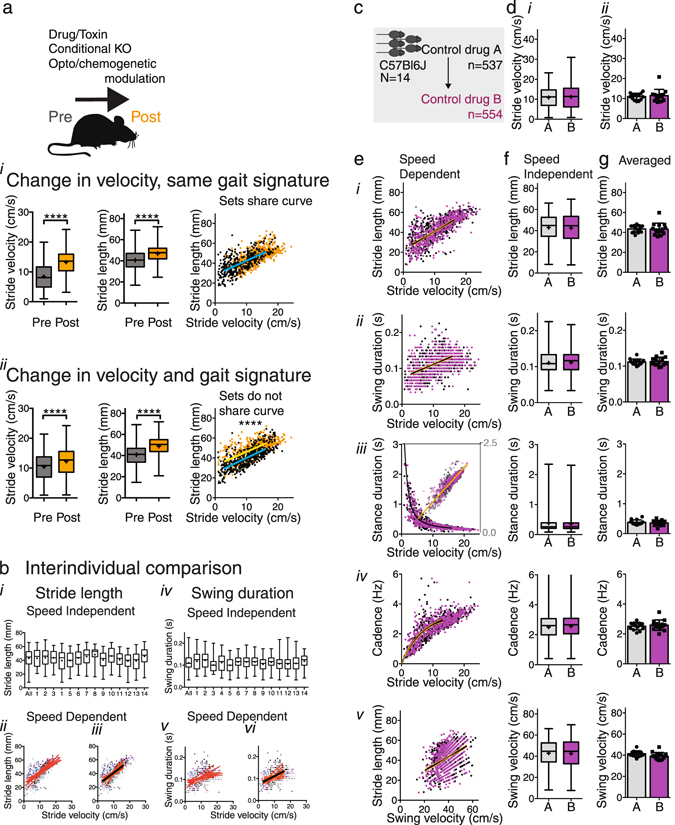
Regression analysis to compare gait datasets during walking. (**a**) Hypothetical datasets showing that regression analysis of stride-to-stride gait data in a velocity dependent manner enables assessment of whether one curve fits both baseline and post-interventional datasets. Changes in gait parameters due to velocity-related changes only do not lead to a shift in regression curve (*i*) whereas changes in gait quality with or without changes in velocity result in a change in slope or Y intercept of the regression curves (*ii*) representing a change in gait signature. Of note, this approach assumes that mice act as their own control or are compared to litter mates. (**b**) Comparison of gait parameters among individual animals within a cohort of C57Bl6/J adult males. *i and iv:* Whisker plots showing little variation between animals(*i and iv*). *Ii-iii* and *v-vi*: Data points (each animal depicted by a different color) and regression curves (orange) represent individual animals for the full velocity range (*ii* and v) and restricted range (*iii and vi*) as in Supplementary Table S2. Black curves represent the shared regression line. Note that regression lines overlap especially for the restricted range. (**c**) Gait parameters were measured in one cohort under 2 control conditions to validate reproducibility. (**d**) (*i*): Box and whisker plots show the distribution of stride-to-stride velocity (center representing median, center cross representing mean, bars representing minimum and maximum; Mann Whitney Rank test, Supplementary Table S3). (*ii*) bar graphs with scatter plots represent average stride velocity (2-tailed t-test), with error bars indicating standard deviation. (**e**) Relationships of stride length (*i*), swing duration (*ii*), stance duration (*iii*), and cadence (*iv*) as a function of stride velocity, and of stride length as a function of swing velocity (*v*) were captured with non-linear regression models (Supplementary Table S2). The fit of one curve to both data sets was compared with the fit of individual curves fit to each dataset (Supplementary Table S3). Orange lines represent 95% confidence intervals. (**f**) (*i-v*) and **g** (*i-v*): Stride-to-stride data represented as whisker plots (Mann Whitney Rank test, Supplementary Table S3; bars indicate maximum and minimum) and bar graphs with scatter plots represent averaged data (paired 2-tailed t-test, Supplementary Table S3; error bars indicate standard deviation).

Analyzing stride-to-stride data sets as a function of velocity has the major advantage that it allows one to assess whether an experimental intervention in mice leads to a change in gait parameters that reflects a) a change in velocity only or b) a change in gait quality or signature (Fig. 2a). Such an analysis can be performed by establishing best fit regression models for each of the gait parameters (Fig. 1e) and then testing whether curves representing datasets from the same animals with or without experimental intervention are shared. To illustrate this approach, we took the baseline stride length data from cohort 4 (C57Bl6J mice; Supplementary Table S1). From this data we generated 2 hypothetical datasets to introduce the concept of shifting curves. The first hypothetical dataset, representing normal gait metrics, contained data at both faster and slower speeds. These data were generated by randomly assigning one quarter of dataset 4 to a faster group and one quarter to a slower group, whereas the remaining half was ranked based upon speed, with the slower half being assigned to the slower group and the faster values to the faster group. This resulted in groups with faster (orange in Fig. 2a-*i*) and slower speeds (black in Fig. 2a-*i*), but with normal gait metrics. For the second hypothetical dataset, to illustrate a change in gait quality, one half of the same baseline stride length data from cohort 4 was not modified (black in Fig. 2A*ii*), whereas 5 mm was added to the stride length of the other half of the dataset (orange in Fig. 2A*ii*), i.e. the gait metrics in the black dataset represented normal gait metrics, whereas the quality of the orange dataset had changed by (artificially) increasing stride length. These datasets then show that if a change in velocity is not associated with a change in gait quality (Fig. 2a-*i*), the pre and post-intervention datasets will share one curve (method applied as outlined below). On the other hand, if stride length increases out of proportion to what is predicted by a change in velocity alone, the gait signature changes and this is characterized by a curve shift from baseline (Fig. 2a-*ii*; with different y-intercept, slope or both; method applied as outlined below). Such information is crucial when studying the effects of CNS modulation on gait in experimental models to distinguish between those models that simply change velocity from those that actually alter gait signatures.

### Regression models that capture gait parameters at walking speed

**Figure 3:**
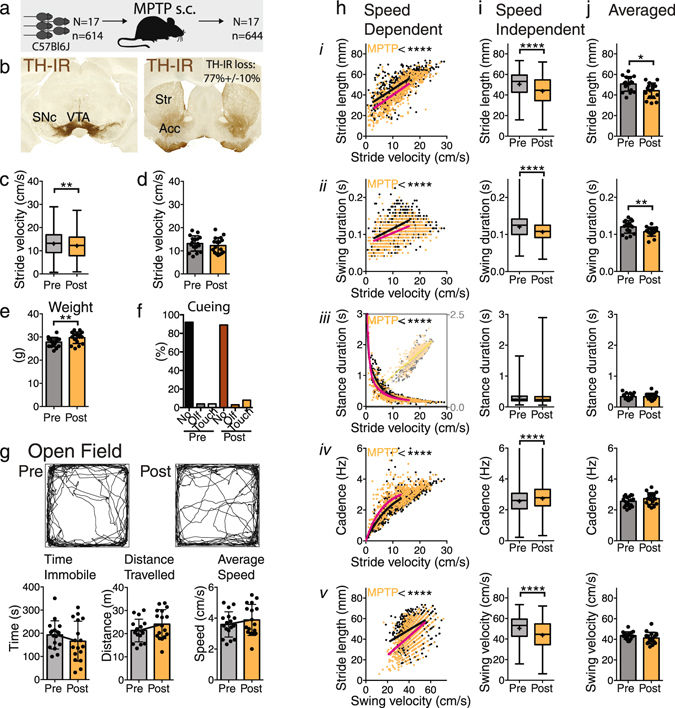
Effects of MPTP treatment on gait. (**a**) In 17 C57Bl6 mice gait and locomotor activity was studied before and after s.c. MPTP treatment. (**b**) MPTP induced bilateral loss of tyrosine-hydroxylase immunoreactivity in the striatum. (**c** and **d**) Velocity was significantly lower post MPTP as determined by stride to stride data (Mann Whitney Rank test; Supplementary Table S3), but this was not detected in the averaged data sets (2-tailed paired t-test; Supplementary Table S3). (e) Post MPTP animals gained weight (paired, 2-tailed t-test). (**f**) Cueing was not necessary to obtain sufficient trials or data points. (**g**) Open field performance was not different pre and post MPTP treatment. (**h**) Relationships of stride length (*i*), swing duration (*ii*), stance duration (*iii*), cadence (*iv*) as a function of stride velocity and stride length as a function of swing speed (*v*). The fit of one curve to both data sets was compared with the fit of individual curves fit to each dataset. All gait parameters showed highly significant differences with shortened stride length, decreased swing and stance duration, and increased cadence (F test, Supplementary Table S3). (**i**) (*i-v*): The distribution of the stride-to-stride data showed significant differences in the same gait parameters as the speed dependent analysis, except for stance duration. However, these are difficult to interpret due to changes in velocity in the stride-to-stride data sets (Mann Whitney Rank test, Supplementary Table S3). (**j**) (*i-v*): Averaged data sets revealed decreased stride length and swing durations, but no changes in other gait parameters (paired 2 tailed t-test; Supplementary Table S3). Asterisks indicate level of signific﻿ance.

**Figure 4:**
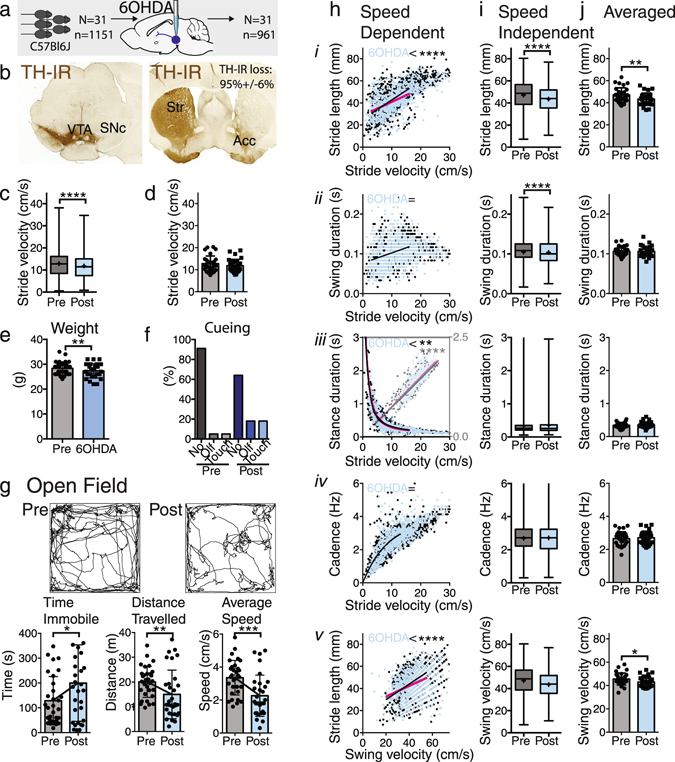
Effects of 6-OHDA treatment on gait. (**a**) In 31 C57Bl6 mice gait and locomotor activity was studied before and after unilateral 6-OHDA injections into the substantia nigra. (**b**) Intranigral 6-OHDA induced unilateral loss of tyrosine-hydroxylase immunoreactivity in the ipsilateral striatum. (**c** and **d**) Velocity was significantly lower post 6-OHDA as determined by stride-to-stride data (Mann Whitney Rank test; Supplementary Table S3), but this was not detected in the averaged data sets (2-tailed paired t-test). (**e**) Three weeks following 6-OHDA, mice had lost weight (paired, 2-tailed t-test). (**f**) When collecting gait data, cueing was used in 40% of the trials. (**g**) Overall locomotor activity in the open field test was significantly reduced and animals spent more time immobile (paired, 2-tailed t-test; Supplementary Table S3). (**h**) Relationships of stride length (*i*), swing duration (*ii*), stance duration (*iii*), cadence (*iv*) as a function of stride velocity and stride length as a function of swing speed (*v*). The fit of one curve to both data sets was compared with the fit of individual curves fit to each dataset. Stride length and stride length as a function of swing speed showed highly significant differences (F test; Supplementary Table S3). (**i**) The distribution of stride-to-stride data showed significant differences for stride length (*i*) and swing duration (*ii*). However, these are difficult to interpret due to changes in velocity in stride-to-stride data sets (Mann Whitney Rank test; Supplementary Table S3). (**j**) Averaged data sets revealed decreased stride length (*i*) as a function of velocity or swing duration (*ii*), but no changes in other gait parameters (*iii-v*; paired 2 tailed t-test; Supplementary Table S3). Asterisks indicate level of significance.

**Figure 5:**
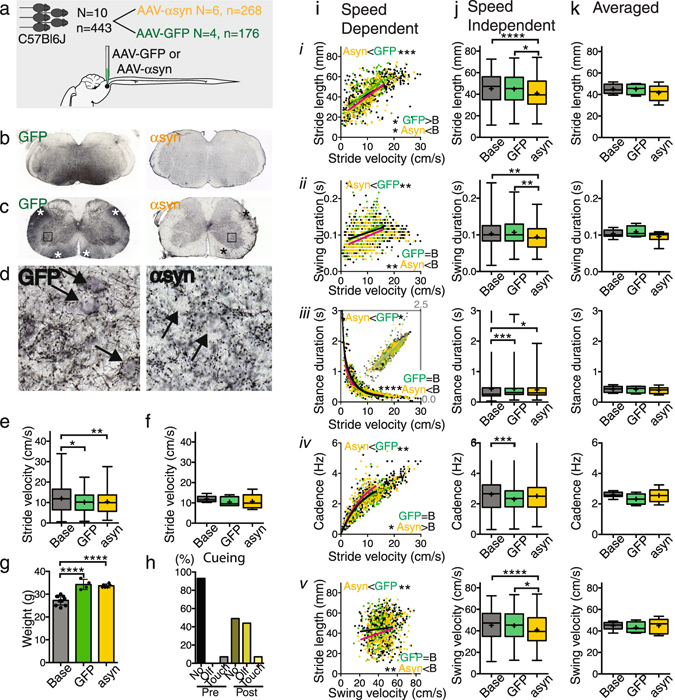
Effects of brainstem alpha-synuclein overexpression on gait. (**a**) In C57Bl6 mice gait was studied before and after AAV-alpha-synuclein or AAV-GFP injections into the mPMRF. (**b**) Immunostaining for GFP (black) shows the injection site, whereas alpha-synuclein immunostaining was not present in cell bodies in the injected region. (**c**) GFP and alpha-synuclein labeled axons in the spinal cord. (**d**) Higher power photomicrographs showing abundant GFP and alpha-synuclein labeled bouton-like profiles in the ventral horn in close apposition to presumed motoneurons (arrows). (**e**) Velocity was significantly lower 18 weeks following GFP and alpha-synuclein injections, as determined by stride-to-stride data, but (**f**) this was not detected in the averaged data sets (Kruskal-Wallis rank test, followed by Dunn’s multiple comparison test; Supplementary Table S3). (**g**) Both in the GFP and alpha-synuclein group mice gained weight (Kruskal-Wallis rank test, followed by Dunn’s multiple comparison test; Supplementary Table S3). (**h**) When collecting gait data, cueing was used in 50% of the trials. (**i**) Relationships of stride length (*i*), swing duration (*ii*), stance duration (*iii*), cadence (*iv*) as a function of stride velocity and stride length as a function of swing speed (*v*). The fit of one curve to each pair of data sets was compared with the fit of individual curves (Supplementary Table S3). (**j**) The distribution of the stride-to-stride data showed similar patterns for stride length (*i*), swing duration (*ii*), and stride length as a function of swing speed (*v*; Kruskal-Wallis rank test, followed by Dunn’s multiple comparison test; Supplementary Table S3). (**k**) Averaged data sets revealed no differences (*i-v*; Mann Whitney Rank test; Supplementary Table S3). Asterisks indicate level of significance.

**Figure 6:**
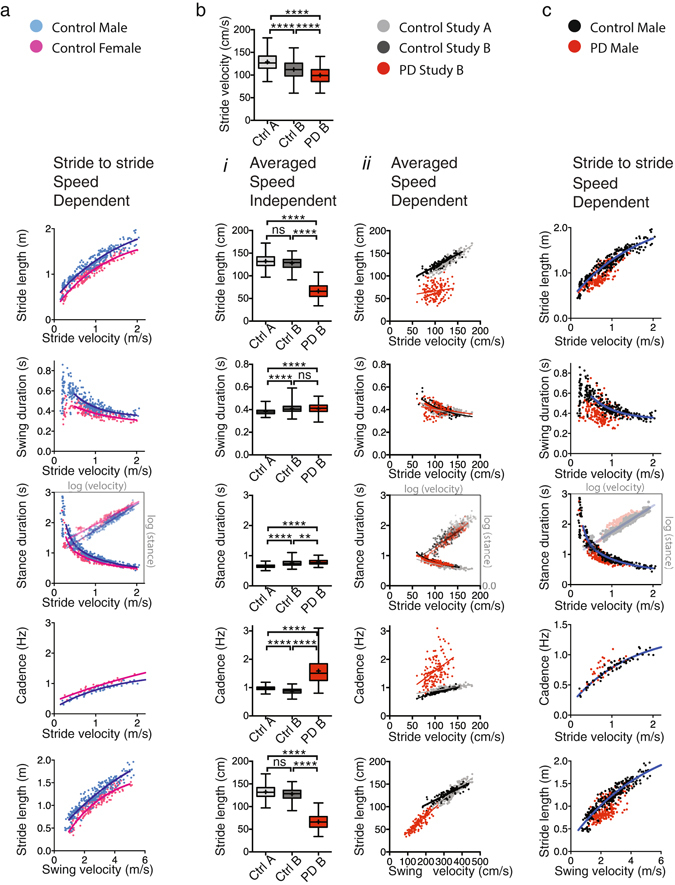
Gait parameters in human control subjects and subjects with Parkinson’s Disease. Spatial and temporal gait parameters depicted as a function of speed in human subjects can be represented by regression curves similar to mice, with regression curves shifting in the setting of CNS disease. (**a**) (*i-v*)**:** Gait parameters visualized as a function of velocity in 14 control subjects without parkinsonism or cognitive decline follow non-linear relationships. Subjects were instructed to walk at velocities ranging from very slow to very fast. Except for cadence which was based upon trial average (*iv*), data points represent stride data of the left foot. Blue represents male and pink represent female data points. Data sets were not adjusted for other biometric or demographic factors. (**b**) (*i-x*): *i-v*, Velocity independent stride-to-stride analysis (whisker plots; Kruskal-Wallis, followed by Dunn’s multiple comparisons test; Supplementary Table S3) shows differences among most gait parameters, which are difficult to interpret given variations in velocity among cohorts. *vi-x*: Data points representing trial averages (from a baseline and a slower trial) in two different cohorts of control subjects (black and gray) and in PD subjects (red) from 2 previously published studies. (**c)** (*i-v*): Data points representing stride-to-stride data (from 6 trials capturing very slow to very fast speeds) in gender and age matched control subjects (black) and PD subjects (red). Asterisks indicate level of significance.

Before comparing datasets, we first need to determine the simplest regression model that best fits each of the gait parameters. From a biological point of view, one would like to establish the gait signature at baseline for a full range of velocities, as illustrated in Fig. 1e. However, this will not be feasible when working with experimental models in which reduction in gait velocity may derive from the intervention being studied and when working with a particular runway size. In addition, gait parameters captured at slow and fast velocities (Fig. 1d) show markedly different velocity dependent relationships and cannot be readily compared. These issues can be minimized by restricting analyses to a specific velocity range. For gait investigations in neurodegenerative disease models and/or for cross species translational studies, the velocity that is most relevant is walking speed, as patients typically present with a variety of characteristic walking problems that further slow their walking. In mice, average walking speed is 9 cm/s with a standard deviation of 6 cm/s^15^ as determined by footfall patterns. Walking range was captured fully on the smaller runway and therefore we used datasets collected on this runway only. In various baseline datasets (Supplementary Table 1), we then applied simple non-linear regression models to empirically determine the simplest preferred model that fits the relationship between each gait parameter and the predictor (speed) among datasets (Figs 3–6, Supplementary Figs S1–2). The parameters that define each of these models are summarized in Supplementary Table S2.

### Validation of gait signatures of normal mouse walking within and among cohorts

Using these models, we then compared the best fit curves within and between age matched mature adult cohorts to assess reproducibility and variation related to sex, genetic background or experimental factors including batch. For each set of experiments, we compared the fit of individual curves to each dataset to the fit of one curve to both data sets combined by an F-test. We have included a step-by-step guide in Supplementary Fig. S3 on how to perform these analyses using Graphpad Prism software for the various types of curves using the parameters summarized in Supplementary Table S2. We contrasted this analysis with results of conventional analyses of stride-to-stride data (Mann Whitney Rank Test or Kruskall Wallis for multiple comparisons) and averaged data (2-tailed paired t-test or ANOVA for multiple comparisons), which do not take into account speed.

#### Within batch

Within a cohort of C57Bl6J male mice (datasets 4 and 5 with control drugs A and B, respectively; Supplemental Table S1; Fig. 2c–g), speed was similar under 2 control conditions. Comparison of curves did not reveal significant differences in any of the gait parameters. Conventional analysis of stride-to-stride data and of averaged data independent of speed also did not reveal any differences (Supplementary Table S3).

Comparing datasets of 2 cohorts of C57Bl6J with similar genetic background, age, sex and shipment (i.e. litter mates; dataset 6 and matched number from dataset 16; Supplementary Table S1; Supplementary Fig. S1; Supplementary Table S3), the curves were also similar among groups. However, as velocity was higher in group B for the stride-to-stride dataset, conventional analyses of stride-to-stride data that did not take into account speed showed differences for all gait parameters except for swing duration. Analysis of averaged data did not reveal group differences.

#### Sex

To introduce known variables, we compared male and female cohorts of mice of the same genetic background and age (datasets 6 and 7; Supplementary Fig. S1; Supplementary Table S3; datasets 7 and 14, data not shown). Stride velocity was the same for both groups, but females weighed less (2 tailed unpaired t-test; t(22) = 10.27, p < 0.0001) and were smaller (nose to base of tail: 59.1 mm + −2.3 mm versus 66.9 mm + −1.8 mm; 2 tailed unpaired t-test; t(22) = 9.24, p < 0.0001). Gait parameters among males and females did not share the same curves, except for swing duration. Stride length and stance duration were shorter and cadence was higher in females compared to males. Conventional stride-to-stride analysis showed similar results as the regression analyses, as expected given that both groups have similar velocities. Analysis of averaged data with a two-tailed Student’s *t-*test did not reveal significantly different stride length, but revealed a higher cadence in females and a higher swing velocity in males compared to females. The smaller size in females likely contributes to the shorter stride length with increased cadence being compensatory when maintaining similar speed.

#### Genetic background

To assess whether genetic background influences gait, we studied 2 cohorts of conditional knockout mice with a mixed genetic background (dataset 8: C57Bl6/J;129P3/J VGaT^fl/fl^ or dataset 9: C57Bl6/J;129P3/J VGluT2^fl/fl^; Supplementary Table S1; Supplementary Fig. S2; Supplementary Table S3). Weights were similar (2 tailed unpaired t-test; t(32) = 0.90, p = 0.38), but the VGluT2 floxed animals were faster. Stride-to-stride, speed dependent regression analysis showed significant differences for swing duration and cadence, but not for other parameters. By contrast, conventional analyses of stride-to-stride data that did not take into account speed yielded different results, with differences in stride length, stance duration and cadence, but not in swing duration. Given the differences in baseline speed between cohorts, conventional analysis results are difficult to interpret. Comparisons of averaged data of each of cohorts with a 2-tailed paired t-test did not show any differences.

#### Batch to batch

Finally, we analyzed 4 baseline C57Bl6J male cohorts, which were derived from different shipments (cohorts c1-c4 from datasets 10, 12, 16 (only data tested by one tester/rater team), and matching number from dataset 14, respectively; Supplementary Fig. S2; Supplementary Table S3). Cohort c3 had a higher weight (One Way ANOVA, p 0.02, F(3,33) = 3.9), and during testing of cohort c2 a male assistant was present (all other cohorts were tested by female staff). Datasets of cohorts c1 and c4 shared the same curves for all parameters, whereas cohorts c2 and c3 showed differences for several parameters. The conventional analyses also showed significant differences between groups, especially cohort c2 and c3.

Altogether, these data indicate that speed-dependent analysis of stride-to-stride data using curve-fitting based upon simple non-linear models is highly reproducible when used within the same group of animals or among groups of littermates under strictly controlled experimental conditions. Conventional analysis that does not take into account speed yields results that are interpretable only when velocities between datasets are the same as assessed using stride-to-stride data.

### Gait signatures in mouse models of experimental parkinsonism

Gait in Parkinson’s disease (PD) is typically characterized by shortened stride length and slower speed. Thus, in this setting it is crucial to differentiate velocity-dependent changes versus shifts in gait signature. It remains unclear whether stride length changes as a function of speed (i.e. gait is simply slower without a shift in curve) or whether the curve representing stride length shifts downward (i.e. stride length is shorter than expected for the particular speed range). For these proof-of-principle experiments, we used two classic mouse models for experimental parkinsonism that result in loss of striatal dopaminergic innervation: systemic administration of MPTP and unilateral 6-OHDA microinjections into the substantia nigra. In addition, given that a) PD gait is only partially amenable to dopamine treatment^24^, b) α-synuclein pathology is prominent in the pontomedullary reticular formation of patients with PD^25^, and c) that the pontomedullary reticular formation is important for locomotion^13^, in a third model we injected AAV-α synuclein into the medial pontomedullary reticular formation (mPMRF) to study effects of α-synuclein overexpression on gait.

#### Model 1: MPTP model

Following subcutaneous MPTP treatment, densitometry of tyrosine hydroxylase in the striatum showed an average bilateral reduction of 77% +/− 10% as compared to an untreated control cohort (n = 8) post-hoc (Fig. 3). Four weeks after MPTP treatment (dataset 17; Supplementary Table S1), velocity was decreased compared to baseline (dataset 16; Supplementary Table S1), though only significantly for stride-to-stride data (Fig. 3c,d). Regression analysis of pre-lesion versus post-lesion datasets from the same animals (Fig. 3h; Supplementary Table S3) showed a downward shift of the curve representing stride length, meaning stride length was shorter than expected for a given speed. Other parameters were also affected: the curve for swing duration shifted downward, stance duration shifted to the left and cadence increased. Conventional stride-to-stride analysis showed significant changes in all gait parameters except for stance duration (Fig. 3i), whereas averaged data showed a decrease in stride length and swing duration only (Fig. 3j). Mice thrived based upon their increase in weight (Fig. 3e; two tailed paired t-test (t(16) = 3.67, p 0.002) and did not require cueing in order to obtain gait data, i.e. had no difficulties with initiation or continuation of gait (Fig. 3f). Measures of the amount of locomotor activity as assessed in the Open Field test with Time Immobile, Distance Traveled and Average Speed, did not significantly change following MPTP treatment (Fig. 3g, Supplementary Table S3). Altogether this suggests that systemic MPTP resulted in a change in quality of gait, without decreasing the amount of locomotor activity.

#### Model 2: 6-OHDA

Unilateral 6-OHDA injections into the substantia nigra resulted in robust unilateral loss of TH-IR from the ipsilateral striatum (Fig. 4b). Similar to the MPTP dataset, stride-to-stride but not averaged stride velocity decreased significantly (Fig. 4c,d; Supplementary Table S3; datasets 10, 12, and 14 for baseline and datasets 11, 13, and 15 for post-lesion effect). Regression analysis demonstrated a change in stride length, but in contrast to the MPTP dataset, the slope rather than the Y-intercept was affected. Stride length was decreased only at higher walking speeds. In contrast to the MPTP datasets, swing duration and cadence did not change, whereas a relatively subtle change was detected for stance duration. Conventional analysis of parameters irrespective of velocity showed a decrease in stride length both for stride-to-stride and averaged data (Fig. 4i,j; Supplementary Table S3). In contrast to the MPTP model, quantity of locomotion as assessed in the Open Field test was affected in this cohort, with increased Time Immobile and decreased Distance Traveled and Average Speed in the Open field test (Fig. 4g; Supplementary Table S3). Also in contrast to the MPTP model, weight decreased following 6-OHDA (t(30) = 2.89; p 0.007; 2-tailed paired t-test); Fig. 4e) and mice required more cueing to start or finish trials (Fig. 4f). Of note, given the severity of the unilateral 6-OHDA lesions, some mice walked in circles (Fig. 4g). In 2 mice, these rotations were so tight that this prevented the collection of any meaningful gait data.

#### Model 3: mPMRF α-synuclein

Following injections of small volumes (30 nl) of AAV-α-syn or AAV-GFP into the medial pontomedullary reticular formation (mPMRF; Fig. 5a,b), post-hoc tissue analysis showed dense α-syn and GFP labeling of axons and terminals extending into the spinal cord (Fig. 5c,d). This indicates that the injections transfected reticulospinal neurons. Baseline gait parameters in the AAV-α*-*syn and GFP control group did not differ from each other using regression or conventional analyses and these datasets were therefore pooled (dataset 18, Supplementary Table S1). After 4 months (datasets 19–20, Supplementary Table S1), velocity had markedly decreased in the AAV-α-syn group compared to baseline, but speed also decreased in the GFP control group (Fig. 5e; Supplementary Table S3), which may have been due to repeated exposures to the runway. As compared to baseline and control GFP groups (Fig. 5i; Supplementary Table S3), the AAV-α-syn group curves for stride length and swing duration shifted downward, stance duration shifted to the left, and cadence increased. The quality of these changes was similar to those seen in the MPTP cohort. Speed-independent analysis showed significant changes in all gait parameters for the AAV-α*-*syn group compared to baseline and control, except for cadence (Fig. 5j; Supplementary Table S3). Weight increased similarly in GFP control and AAV-α-syn groups (Fig. 5g). In contrast to the MPTP group, the AAV-α-syn group required significant cueing to start or finish trials (Fig. 5h).

Summarizing, these datasets show that the different experimental mouse models of parkinsonism affect diverse aspects of gait and locomotor activity. Systemic MPTP and α-syn mPMRF models affect gait signatures, whereas the 6-OHDA model has relatively subtle effects on gait signatures but has large effects on locomotor activity. Thus, a single experimental mouse model insufficiently describes the diverse impairments that together constitute the full range of motor impairment in parkinsonism, due to different pathologic processes or focal CNS lesions underlying the various impairments.

### Graphical summaries of gait metrics in human control subjects show tight velocity dependent relationships similar to mice

Next we determined whether spatial and temporal gait parameters at walking speed in human subjects behave as hind limb datasets in mice. We collected stride-to-stride data from different trials that covered a wide range of human walking speeds (Fig. 6a,b). In contrast to mice, human subjects are able to maintain a steady velocity and can modulate velocity between trials when instructed to do so, leading to clustering of data points for each of the trials. Data from 14 older healthy adults shows that spatial and temporal gait parameters are tightly linked and vary with walking speed as observed in mice. While these gait parameters show velocity dependent relationships in both mice and human there are also cross species differences. For example, swing duration is increased at lower velocities in humans as compared to mice. Best-fit curves, without statistical inferences, illustrate non-linear relationships for the full walking range. Curves of male (blue) and female (pink) subjects, with similar age, weight and cognitive function, but lower female height (p < 0.0001; two tailed *t-*test) ran parallel to each other. Given the variability among human subjects including age, height, weight, overall fitness, and other demographic variables, more complex multilevel modeling^23^ or mixed regression models using larger datasets will be required for optimal fully adjusted modeling. However, the current data show that human gait parameters visualized as a function of velocity behave similar to mice datasets, and thus are suitable for further modeling.

### Gait signatures in human subjects with and without Parkinson’s disease

Given that gait parameters form characteristic velocity dependent signatures during walking both in mice and human subjects, we then questioned whether gait curves that capture these relationships deviate in PD similar to one or more of the mouse models. To explore this possibility we used data from two published studies (Fig. 6b ^3, 4^), including control cohorts A and B, and PD cohort B. Average velocity was greater in control group A than B, whereas both control groups walked faster than the PD group (Fig. 6b). The distributions of the gait parameters plotted as a function of velocity in the control groups (black and gray in Fig. 6b) strikingly overlapped, especially given that these were not corrected for variables such as age, sex, height or demographics. By contrast, data from PD subjects (red; Hoehn and Yahr 2 or 2.5; Fig. 6c) was strikingly different for some, but not all gait parameters, compared to age and sex matched controls. There was a marked decrease in stride length and an increase in cadence, whereas datasets representing stance duration overlapped. As for swing duration, control and PD datasets also overlapped except for a cluster of data points with shorter swing time than expected, derived from a subgroup of subjects. We did not apply formal statistical regression analysis as obvious variables (such as sex, age, height and leg length) would not allow for simple regression models (as used in mice) and given that the sample size was too small to take these variables into account in more complex models. Conventional non-speed dependent analysis of the control and PD datasets showed differences for many gait parameters, which in themselves are difficult to interpret given the differences in average speed among groups.

## Discussion

We present an approach that captures the unique signatures of spatial and temporal gait parameters as a function of walking velocity in mice and humans. This approach has several important advantages over conventional methods. First, it provides a powerful way to visualize and summarize gait parameters, which aids interpretation of complex datasets. Secondly, it allows detection of changes in different aspects of walking irrespective of whether these changes occur with or without differences in walking speed. This latter point is particularly relevant as many disease models are accompanied by a change in walking speed. Finally, given the similarities between mice and human models, it allows for comparison of shifts in gait signatures between animal models (from baseline to experimental) and in human datasets (from control to disease condition to medical intervention). This opens up novel translational opportunities which may help elucidate CNS circuits involved in the control of walking, select or develop appropriate disease models, monitor progression, and evaluate efficacy of medical, cognitive, exercise or surgical intervention, both in mouse models and a wide range of clinical settings.

High speed video analysis or other methods of ground walking foot print analysis have become increasingly popular and accessible in rodent studies, including not only circuit mapping studies that focus primarily on locomotion, but also translational studies in which gait changes form one of the outcome measures. For analysis, data sets are often averaged which, as shown in this study, impedes full characterization of the velocity dependence and non-linear nature of gait parameters. Only if stride-to-stride data shows that there is no change in speed, such a simple comparison of parameters is valid. If speed differs among groups, it becomes challenging to interpret whether gait parameters change in relation to a change in speed or not. This contributes to conflicting results even in straightforward, classical experimental PD models^26–30^. More complex, regression-based analysis approaches have been used to take the velocity dependent, non-linear gait characteristics into account to compare ground and treadmill running^16^, in experimental models of spinal cord injury^17, 18^, following Purkinje cell degeneration^20^, or in knockout models that result in changes in spinal circuits^19^. In this study, we applied a regression-analysis based approach to develop a robust method that captures changes in gait signatures at *walking* velocities, the velocity range that is most relevant for translational studies. This method is highly reproducible under the appropriate experimental conditions, i.e. when using the same mice or littermates as controls. Even without regression analysis, visualization of stride-to-stride gait data in a speed-dependent manner is crucial for assessment of the quality of the data, and can be applied to any stride-to-stride dataset, as we demonstrated in the human cohorts.

To overcome the issue of gait parameters being velocity dependent, the treadmill method poses another alternative. This approach, however, does not fully overcome the speed issue as it prohibits measurement of preferred speed, which may be the hallmark of a phenotype. This method also introduces uncertainty as to what speed(s) to set the treadmill. For example, speeds of 16 to 18 cm/s, above the range analyzed in this study, did not reveal differences in gait parameters in the 6-OHDA model^31, 32^. In an MPTP model with treadmill speed at 34 cm/s, gait parameters changed similar to our study^33^, but changes were not detected with the treadmill set at 10 cm/s. In addition, over-ground walking does not require as extensive training as the treadmill, and training ability is a potential confounder especially when studying supraspinal circuits. In addition, both in mice and humans, kinematics and kinetics during ground and treadmill walking are different^16, 34^. Analysis of patterns of footfall^15^ pays full attention to changes in velocity and is another helpful tool to understand changes in locomotor patterns, although such patterns are more challenging to translate between quadruped and biped models. At higher speeds, quadruped animals show increasing frequency of gait patterns such as trot and gallop which are typically not part of human bipedal gait patterns.

The approach we developed has several limitations. It is two-dimensional, developed specifically to compare effects of interventions within one cohort or between tightly matched cohorts (i.e. litter mates), the most common and preferred way to control experimental conditions in animal studies. This approach may not be suitable to derive a unifying curve for one entire species or strain, to make predictions based upon the model, or to determine effects of experimental intervention in unmatched cohorts, in case of significant inter-animal differences. A mixed-model approach^20^ would be required to estimate contributions from such inter-animal differences or differences due to variations in experimental conditions. For this reason, we did not apply the simple models to human datasets. Within human datasets, given clustering of trial data and obvious inter-individual variation due to known and unknown factors, larger datasets will be necessary to develop appropriate mixed models or multilevel models^23^ that allow quantitative comparisons.

The approach is sensitive to differences in experimental conditions, ranging from cohort matching to testing conditions to trial selection to runway size. As with any behavioral test, tight control of experimental conditions is therefore crucial. We focused on a slower velocity range, for the purpose of translation. For datasets with different ranges of speed, the models would need to be adjusted. Also, we focused on a restricted set of gait parameters. Whether regression analysis is necessary for other gait parameters remains unclear especially as not all gait parameters are velocity dependent.

Furthermore, the current analyses included combined data of the left and right hindlimbs in the mouse studies, and data of one side in the human studies. Therefore, relevant asymmetries in temporal or spatial gait metrics between the left and right side in the 6-OHDA model, or asymmetries between least and most affected side in the human studies may have remained undetected. Additional analyses in future studies will be necessary to capture such asymmetries. Finally, we did not adjust levels of significance for multiple comparisons or multiple measurements. For a conservative use of this approach, a p level of 0.001 will still yield similar results, while avoiding over-interpretation of results.

Visualization of hind limb gait parameters as a function of speed shows striking similarities among quadruped mammals from elephants^35^ to mice^19^ and in biped humans, despite obvious differences in size and musculoskeletal anatomy between these species. This supports the hypothesis that it is not the size or the anatomy of the musculoskeletal system that underlies the gait signatures which we have derived. Instead, it is likely the CNS that sets these signatures through activation of spinal pattern generator networks that are relatively conserved evolutionally. Interventions to CNS circuits indeed disrupt or modulate the relationships of gait parameters as a function of velocity, whether at the spinal level^17, 19^ or supraspinal levels as shown in the current study. Additionally, these disruptions are present in both animal models of parkinsonism and human PD.

Though the similarities between gait signatures among species are striking, they are not the same. This is most obvious for swing time, which deflects upward at lower speeds in human subjects (Fig. 6a), whereas it deflects down in mouse datasets (Fig. 1d). This may reflect a true difference in CNS control of swing time, but may also reflect that subjects slowed their speed under conscious control whereas this occurred spontaneously in mice (with unknown internal or external cues). Similarly, various external factors may modulate gait curves. For example, stride length is limited by leg length, which may explain the different stride length curves in female and male mice and humans. The sex related differences in mice are in line with weight related changes measured in a prior study in mice^20^. To directly test whether there body size is to sole factor contribution to the sex differences that we measured in spatial and temporal gait parameters, further experiments will be necessary.

An important implication of our results in PD models is that changes in gait signatures can be dissociated from changes in locomotor activity. Supraspinal circuits are important for initiation of locomotion, change in direction, and control of velocity^13, 36^. The current study demonstrates that supraspinal circuits additionally modulate gait signatures, with or without changes in overall locomotor activity or speed. The amount of locomotion (i.e. how much walking is done) and modulation of gait signatures (i.e. what does the walking look like) are not necessarily mediated by the same circuits. Separate neural circuits may modulate the quantity and speed of locomotion in such a way that the natural speed dependent properties or signatures are being maintained (i.e. parameters stay on the curve), whereas other circuits serve to adjust the quality of gait leading to a change in gait signature, with gait parameters changing out of proportion to speed. Ongoing studies will elucidate which supraspinal populations modulate each of the gait parameters versus velocity only, in which direction, and how they interact.

A universal gait analysis approach provides opportunities for translation. Visualization of stride-to-stride data sets provides an opportunity to translate findings between species, especially when it comes to disease models. Deviations of distinct sets of gait curves in experimental disease models differentially resemble deviations observed in PD versus age and sex matched controls. For example, curve changes due to systemic MPTP or mPMRF α-synuclein resemble the direction and degree in gait curve shifts in Parkinson’s disease more than the substantia nigra specific lesions of the 6-OHDA model, while the latter model significantly affects overall locomotor activity. These results suggest that non-dopaminergic circuits are important contributors to changes in curve shifts (i.e. changes in gait signature rather than gait speed alone). From a clinical point of view, this fits with the notion that not all features of PD can be ameliorated with dopamine therapy^37^. Moreover, abundant α-synuclein pathology is present in the mPMRF^25^ in postmortem tissue of subjects with PD, homologous to the site we targeted by α-synuclein overexpression in mice. With conditional knockout models and opto- and chemogenetic tools we are currently identifying the relevant mPMRF circuits in mice. This information can then be translated back to human conditions, and tested in a hypothesis-driven way in human subjects, i.e. with functional imaging, advanced electrophysiological approaches and/or clinical-pathological approaches.

In addition, visualization of gait parameters in human patients can be used to monitor progression of gait decline over time, to study gait signatures in different genetic or clinical subtypes of PD, to assess the interaction between weaknesses in cognitive domains and shifts in gait signatures, or to quantify effects of interventions with medications, exercise or deep brain stimulation on gait quality and speed. In summary, the methods described here have the potential to quantify qualitative walking abnormalities related to CNS circuit dysfunction across species, can be used to help identify appropriate animal models, and open up important translational and clinical opportunities.

## Methods

### Animal data sets

Handling and housing of animals, surgical procedures, post-operative monitoring, and behavioral tests were performed in accordance with the Guide for the Care and Use of Laboratory Animals at the animal research facility of the Center for Life Sciences at Beth Israel Deaconess Medical Center and at the NeuroBehavior Laboratory at Harvard Institute of Medicine. The experimental protocols were reviewed and approved by the IACUC at BIDMC or Harvard Medical School.

We used 20 datasets (from a total of n = 179 mice; Supplementary Table S1) to assess the effects of: experimental setup (Datasets 1 to 3), within (4 and 5) and between (6, 8, 9, 12, 14 and 16) group variability, sex (6 and 14 versus 7), genetic background (8 and 9), and experimental intervention in 3 models for Parkinson’s disease (10 to 20). In addition, we generated two hypothetical datasets from dataset 4 to introduce the concept of shifting curves.

C57Bl6J mice were shipped from Jackson labs at the age of 8–10 weeks, whereas VGaT^fl/fl^ and VGluT2^fl/fl^ mice^38, 39^ were bred at BIDMC. Mice were group housed (3–5 mice/cage) and in the mature adult age range when tested. Power calculations with relevance for each of the gait parameters could not be performed a priori given the novelty of the approach. Minimum group size for the experimental intervention models was therefore estimated upon power calculations derived from locomotor measures in the open field test (distance traveled in 10 min in the 6-OHDA group). A sample size of 8 would be required based upon a mean of 21.4 + −7.2 m prior to intervention to 11.6 + −6.5 m following intervention, to detect an effect with an alpha error of 0.05 and power of 0.95 using a paired 2 tailed t-test. For the experimental part of this study (datasets 10–20), mice were not randomized but served as their own control. Except for cohorts 18–20, all cohorts formed baseline or control groups as part of studies in which we manipulated the CNS either through selective loss of neurotransmitter signaling from the pontomedullary reticular formation (Datasets 1,2, 8 and 9), or chemogenetic inhibition of the subthalamic region (4–17). The results of these manipulations on motor behavior and gait will be reported separately.

### Induction of parkinsonism

To induce parkinsonism, in 3 cohorts (n = 31) we injected 6-hydroxydopamine (3 μg/μl 6-OHDA, Sigma, and 0.2 mg/mL ascorbic acid in sterile saline; total volume 450 nl; adapted from ref. 40) unilaterally into the substantia nigra via a glass micropipette using a Kopf stereotactic apparatus. Surgery was performed under ketamine/xylazine (100 mg/kg and 10 mg/kg, respectively, i.p.) anesthesia and under aseptic conditions. Animals received meloxicam (5 mg/kg) s.c. peri-operatively for 48 hours. We tested these mice prior to induction of parkinsonism (datasets 10, 12, 14) and 3–5 weeks after induction (sets 13, 15, 17).

In another cohort of 17 mice, we subcutateneously injected 1-methyl-4-phenyl-1,2,3,6-tetrahydropyridine (MPTP; Sigma; 25 mg/kg body weight for 5 consecutive days^41^. We assessed gait prior to (set 16) and 4 weeks after induction of parkinsonism (set 17).

In an additional 10 mice, 30 nl of AAV5-CBA-α-synuclein (titer: 1.0 10e13; UNC Vector Core; Michael J Fox Foundation) or 30 nl of AAV5-CBA-eGFP (titer: 9.5 10e13; UNC Vector Core; Michael J Fox Foundation) were injected into the medial pontomedullary reticular formation under isoflurane anesthesia (2–4% in 100% O2) and aseptic conditions, using a posterior approach to avoid the cerebellum^42^. We assessed gait prior to surgery and up to 4.5 months following the AAV injection.

### Behavioral data collection

All cohorts, except for group 2, were tested on custom made acryllic runways with dimensions of 90 cm × 6 cm^19^. This setup features a mirror underneath the runway placed at a 45 degree angle. This allows for simultaneous visualization of paw placement from the side and bottom, increasing accuracy of gait scoring (see below). Animals were habituated to the runway for at least 2 sessions on non-consecutive days. Testing was done during the first 4 hours of the Light Phase (8.00–20.00) and on non-consecutive days only. Weight was measured on testing days. Gait was recorded using Casio Exilim cameras (frame rate of 120 frames per second, shutter time 1/1000 sec, AVI format) from the middle 30 cm of the runway, for 4–8 trials per mouse and condition, which was sufficient to capture a minimum of 4 trials that each contained at least 3 strides. For each mouse, these trials were obtained within a time frame of 5–15 minutes. For within-group reproducibility experiments, the same cohort was re-tested in a similar fashion at a 1 week interval (datasets 3 and 4, Supplementary Table S1). Videos were loaded in Matlab, and analyzed frame-by-frame using custom made software which corrects for mirror and lens distortion. Each video was calibrated for spatial dimensions using markers on the runway. Per mouse and condition, 4 trials were analyzed in which mice walked for at least 3 strides, excluding starting and stopping steps. For each paw, the start and end of the swing phase with the corresponding paw location was marked. From these parameters we calculated stride velocity, stride length, stance duration, swing duration, stride duration, cadence, swing speed, and base width. Data representing left and right limbs was grouped. With the exception of cohort 2, gait data was scored by raters who differed from the testers, and who were blinded to cohort and/or condition, though abnormalities in some of the PD models were obvious to the tester and scorer.

As for the resolution and validation of this system, placement of the paw on the runway was captured by assigning a pixel (space) to a video frame (time). This data point was derived from two points of vision: a lateral view allows for determination of the frame that closest represents the start or end of the swing phase, whereas a mirror at 45 degrees allows visualization of the position of the paw during stance. The smallest detectable change in space depended on the resolution of the video and calibration of spatial dimensions. Based on 205 calibrations, the average spatial resolution is 0.276 +/−0.0057 mm/pixel. The spatial metrics (stride length) obtained using this analysis system were similar to stride length measures obtained using the paint footprint analysis method^43^ in the same mice. The temporal resolution of the system is dependent on the frame rate of the camera, i.e. 1/120 second (or 0.0083 seconds). Higher frame rates would allow for detection of smaller differences related to swing duration irrespective of speed and for stance duration at higher speeds. A shutter time of 1/1000 second avoided blurring of the frame during faster movements. Data sets obtained using this system overlapped with the data obtained using the Cleversys runway system (see below) and were in the same range as other studies in the mouse^16, 18–20^.

In the experimental PD cohorts, mice had variable degrees of difficulty making it across the runway in a time-window feasible for high speed video data capturing. Olfactory cueing with home cage bedding or tactile cueing was necessary to complete a trial, and these trials were marked as such. We analyzed uncued and each of the cued data sets separately. As touch cued data resulted in erratic measures, we omitted this data from final analyses. Olfactory cued trials were only included if insufficient non-cued trials could be obtained, and we determined the percentage of uncued, olfactory cued and touch cued data in these cohorts.

In cohort 2, we used a larger runway (Runway, Cleversys Inc, dimensions 165 cm × 20 cm, with “home” boxes at either side of the runway). We habituated mice as above. Once the mouse entered the view of the camera, which included the middle 40 cm of the runway, the camera started automatically and recorded for 30 seconds at 80 frames per second (AVI format). We collected 4 trials per mouse, scored it using RunwayScan Software (Cleversys), followed by a manual check of all trials, and extracted the stride-to-stride gait data from the raw data files for further analysis. The temporal resolution of the Cleversys dataset was lower than for the custom setup given the lower video frame rate (1/80 second), whereas the spatial data was comparable to the custom setup.

In the 6-OHDA and MPTP cohorts we determined overall locomotor activity using video tracking during an open field test. Following habituation on non-consecutive days, mice were tested for 10 minutes in a by 50 cm by 38 cm box before and after induction of parkinsonism. We analyzed these data using Anymaze software (Version 4.98, Stoelting, Wood Dale, IL, USA), and extracted measures of total distance travelled, average speed and time immobile.

### Behavioral data analysis

#### Visualization

We visualized gait parameters as stride-to-stride and averaged data, both in speed independent and speed dependent manners. Stride-to-stride datasets were represented in whisker plots, indicating minimum, 25^th^ percentile, median, 75^th^ percentile and maximum. In addition, average data of each of the animals were depicted as bar graphs with scatter plots. To visualize stride-to-stride data in a velocity dependent manner, we plotted each of the gait parameters as a function of the accompanying stride velocity (Fig. 1).

#### Regression models

We used simple non linear regression to assess which model best captured the relationship between gait parameter and the predictor (speed) by using Extra sum-of squares F test (Equation 1). In case of a fit, the simplest best fit model was determined using a F-statistic with a corresponding p-value ≤ 0.05 considered statistically significant, which implies one curve is preferred over the other.

1 $${\rm{F}}=\frac{({\rm{SS1}}\mbox{--}{\rm{SS2}})/({\rm{DF1}}\mbox{--}{\rm{DF2}})}{{\rm{SS2}}/{\rm{DF2}}}$$

SS, sum of squares error; DF, degrees of freedom.

We empirically tested linear, one phase and two phase associations. This was done for the full velocity range and then for limited data ranges (segments) by systematically omitting increasing ranges of the slowest velocities (starting at a bin of 0–1 cm/s and increasing by 1 cm/s) and/or the highest velocities (starting at >25 cm/s and decreasing by 1 cm/s). The rationale of the latter approach was to determine the simplest model that adequately captures the data near the range of walking velocity (average 9 +/− 6 cm/s^15^ in baseline datasets that represent different cohorts, sex, or strains. Omitting the very slowest velocity range (0–3 cm/s) and the higher velocity >16 cm/s resulted in models that were reproducible among baseline cohorts. Using the simpler model if linear and one phase or if one phase and two phase associations fit a dataset reduced the problem of detecting statistically significant changes between datasets that are not relevant from a physiologic point of view.

In many cohorts, the preferred models also fit wider ranges of velocities, but the restrictions in velocity range resulted in more appropriate fits based upon the distribution of residuals and/or standard errors. The Run’s test remained not significant (i.e. model fit the data) whether ranges were restricted or not. However, despite the restricted velocity range, the residuals of the datasets did not always meet the normality test (D’Agostino and Pearson Omnibus K2). Through visualization of residual plots, the Y axis appeared balanced, there were no nonlinear trends, and extreme outliers were not present, but the X-axis remained unbalanced at variable degrees for the various gait parameters. Only severely restricting the range on both ends up to 8–12 cm/s (which also lowered the data points in the sample) would resolve this, but this defeats the purpose of studying gait in the full range of walking velocity. Given that this feature was not related to one cohort but is inherent to gait parameters, and given that we will be using this approach for comparisons within cohorts rather than to make predictions from one to another cohort, ranges were kept liberal to better reflect the data of interest.

Given that each animal is represented by multiple data points, clustering of individual subject data due to characteristics of these groups could skew the results and if so, make the model invalid. It should be stressed that the main goal of the above approach is not to establish a best fit model that represents a universal curve for each gait parameter in mice, to use the model to predict values, or to compare unmatched cohorts, but to develop a model that allows for comparisons among baseline/control and experimental conditions either in the same animals or in precisely matched animals. Therefore this is less of an issue as mice are used as their own control under strictly controlled experimental conditions. However, this can be relevant when baseline and experimental groups represent different mice. We therefore examined inter-individual variability among adult, male C57Bl6J mice from the same age and shipment, representative for a typical dataset (Fig. 2b). The distribution of temporal and spatial gait parameters was homogeneous whether examined in a speed independent or speed dependent way. Under these strict conditions, a more complex model that corrects for such inter-animal variability does not add value. We will assess under which experimental conditions a simple model is no longer valid and would require more complex mixed models.

After establishing the best fit model for each of the gait parameters and the appropriate corresponding velocity range, we then compared datasets from mice with a range of variability in experimental conditions to determine under which experimental conditions the approach is valid. We used an F-statistic (Equation 2) to compare the fits of separate curves for each data set with the fit of a single curve to fit both data sets, i.e. the total sum of squares and the degrees of freedom from the two fits (done separately), compared with the sum of squares and degrees of freedom obtained by combining the data. The F ratio and P value determine if one curve fits both datasets. A p-value < 0.05 was considered statistically significant, which implicated the datasets were so different that they were best plotted as two separate curves. We will discuss whether adjustments of this level are warranted based upon the properties of the datasets.

2 $$F=\frac{({\rm{SScombined}}-({\rm{SSgroup}}\,{\rm{A}}+{\rm{SSgroup}}\,{\rm{B}}))/(\mathrm{DFcombined}-(\mathrm{DFgroup}\,{\rm{A}}+{\rm{DFgroup}}\,{\rm{B}}))}{({\rm{SSgroup}}\,{\rm{A}}+{\rm{SSgroup}}\,B)/({\rm{DFgroup}}\,A+\mathrm{DFgroup}\,{\rm{B}})}$$

SS, sum of squares error; DF, degrees of freedom.

We applied this approach to the same mice under control conditions with the same tester and rater (sets 4 and 5), groups of mice from the same genetic background, shipment, age and sex (i.e. littermates) and with the same tester but different raters (sets 6 and 7), groups with different sex, but the same genetic background, age, tester and rater (sets 6 or 16 and 7), different cohorts from a mixed genetic background, but with the same age, sex, tester and rater (sets 8 and 9), and different cohorts of mice from the same genetic background, age and sex, but with different tester or rater and from different shipments (groups 10, 12, 14, 16).

For conventional analyses that do not take into account variations in speed, we tested stride-to-stride and averaged datasets for normality using the D’Agostino and Pearson test. Sets of normally distributed data were compared using two tailed unpaired or two-tailed paired *t-*test (as appropriate), whereas non-normally distributed datasets were compared using Mann Whitney Rank test. In case of more than two sets, we used one-way ANOVA followed by Holm-Sidak multiple comparison test, or, for non-normally distributed data Kruskal-Wallis rank test followed by Dunn’s multiple comparison test with a family wise level of significance set at p = 0.05.

#### Application to disease models

For the 6-OHDA and MPTP datasets, in which each mouse acted as its own control, we used the same regression and conventional analyses approach as for the validation study. For the AAV-α-synuclein experiments, we used a Kruskal-Wallis test followed by Dunn’s multiple comparison test to compare combined baseline, GFP control and experimental, α-synuclein groups, with a family wise level of significance set at 0.05. The baseline control and experimental groups were combined to yield more power, as group comparisons did not reveal differences between these groups at baseline.

For open field datasets in the MPTP and 6-OHDA experiments we tested the data sets for normality (D’Agostino Pearson test) and then compared baseline and post-lesion data using a two tailed paired t-tests.

All analyses were performed using Graphpad Prism 6 or 7.

### Tissue processing and analysis

Following behavioral testing, we transcardially perfused the mice with PBS followed by 10% formalin as described previously^44^ under chloral hydrate anesthesia (500 mg/kg i.p.). Brain tissue was cut in 4 series of 40 µm sections using a freezing mictrotome. In the MPTP and 6-OHDA groups, one series of free floating sections was processed for tyrosine hydroxylase (TH; sheep, AB152, 1:5000; Lot 2668078; Millipore; Antibodyregistry.org) using a standard immunohistological protocol^44^. In the AAV group, one series was processed for to detect human α-synuclein (LB509, 1:1000; DAKO, Lot CTJ1556^45^; Antibodyregistry.org) and control tissue was processed for GFP (rabbit, A6455; serum; 1:20,000; Invitrogen; Antibodyregistry.org) or AB11122 (IgG fraction, 1:20,000; Invitrogen; Antibodyregistry.org). Following incubation with the primary antibody overnight, tissue was processed with the appropriate biotinylated secondary antibody (1:500) for 2 hours (Jackson Immunoresearch: donkey anti-rabbit, anti-sheep, or anti-mouse) followed by an avidin-biotin peroxidase step (ABC Elite, Vector Laboratories; 1:500). Immunostaining was visualized with 3,3′-diaminobenzidine (Sigma; 0.01% with or without nickel ammonium sulphate) in 0.1% H202. Sections were mounted onto glass slides, dehydrated, cleared and coverslipped with Permaslip mounting medium.

We digitized slides containing the substantia nigra and striatum of all animals in the 6-OHDA and MPTP cohorts (Hamamatsu Nanozoomer XR; 20x magnification). We selected 4 sections representing different levels of the striatum. Using FIJI software, images were calibrated using a step-tablet, and we then calculated optical density using the Rodbard function. Optical density of left and right striatum was taken, and the optical density of a neutral area (neocortex) was subtracted from these values. For the 6-OHDA datasets, we included animals with a > = 80% decrease in TH-IR density compared to the control side as established prior to analysis^40^(31 out of 45; average loss 95% +/− 6%), whereas animals included in the MPTP group had a combined bilateral loss in TH-IR of > = 65% (17 out of 19; average loss 77% +/− 10%). Brainstem and spinal sections of all animals in the AAV experiments were digitized as above. In 6-OHDA, MPTP and AAV cohorts, staining was reproducible and representative images were selected for Figs 3, 4 and 5 respectively.

### Human data sets and analysis

The goal of the human data sets was to assess whether representing human gait data obtained via various technologies yields patterns similar to mouse data sets under control and disease conditions. We used data sets from two previously published studies, including control subjects (study 1^4^) and subjects with Parkinson’s disease and matched controls (study 2^3^), as well as data that was prospectively gathered (ClinicalTrials.gov ID 2015P000310). These studies were approved by the Human Studies Committee at Tel Aviv Sourasky Medical Center or the Institutional Review Board at Beth Israel Deaconess Medical Center and carried out in accordance with institutional guidelines. All subjects provided written informed consent prior to enrollment in the studies.

From study 1, we analyzed the data of healthy older adults (n = 28, age 69.8 ± 6.3yrs, height 1.69 ± 0.07 m, weight 72.7 ± 12.5 kg). From study 2, we used the data of subjects with Parkinson’s disease (n = 36, Hoehn and Yahr stage 2 or 2.5 (2.10 ± 0.2), age 61.22 ± 8.98yrs, height 1.68 ± 0.07 m, weight 73.75 ± 11.84 kg, 23 male, 13 female) and matched healthy subjects (n = 30; age 57.70 ± 6.96yrs, height 1.68 ± 0.09 m, weight 74.31 ± 12.52 kg, 18 male, 12 female). Only ground walking trials without assistive devices were included in our analysis. Both studies used a computerized system to quantify gait by measuring forces underneath the foot as a function of time, as previously described^3, 4, 46, 47^. The system consisted of a pair of shoes and a recording unit. Each shoe contained eight load sensors (capacitive sensors with an accuracy of 2% FSO; force range 0–1000 N/sensor; resolution 2.5 N) that covered the surface of the sole and measured the vertical forces under the foot. The recording unit was carried on the waist. Plantar pressures under each foot were recorded at a rate of 100 Hz. Measurements were stored in a memory card during the walk, and, after the walk, they were transferred to a personal computer for further analysis. Stride duration, swing duration, and swing percentage were determined from the force record. Average gait speed per trial was determined using a stopwatch by measuring the average time the subjects walked the middle 8 meters of a 20 meter (study 1), or the middle 10 meters of the 35 meter walkway (study 2) during two minutes of testing. Average trial stride length was calculated by multiplying the gait speed of the subject by the stride duration of the subject^3^. From these parameters, we calculated trial averages for cadence, stance duration, a log transformation of stance duration and swing speed. Gait parameters represented one average per subject per trial and we used 2 baseline ground-walking trials per subject in Study 1, and two ground-walking trials from Study 2, one at preferred speed and one at slower speed. Datasets in both studies represented gait metrics from the right leg. We plotted the data sets similar to the mouse data sets.

To obtain stride-to-stride datasets covering the full walking range, better matching the mouse datasets, in 14 control subjects (9 male, age 62.6 +/− 7.0; height 175.5 +/− 5.0 cm; weight 88.6 +/− 7.7 kg; MoCA 28.3 +/− 1.6, range 25–30), 5 female; age: 66.8 +/− 9.2; height: 161.5 +/− 2.9 cm; weight: 75.6 +/− 22.9 kg; MoCA 28.0 +/− 2.3, range 25–30), we captured spatial and temporal gait data using a gait mat (Zeno Walkway, Protokinetics, Havertown, PA, USA; 16 × 2 feet; 576 pressure sensors per square foot; sampling rate 120 Hz; spatial resolution 0.5 by 0.5 inches; temporal resolution 1/120 second) and PKMAS Software (Protokinetics). PKMAS software has been validated against the GaitRite software^48^, and the PKMAS/Zeno system has also been validated against force plates measuring center of pressure during walking^49^. Furthermore, the Zeno Walkway, formerly known as “GaitRite MSQR”, and GaitRite system have been validated against Clinical Stride Analyzer^50^ and a video system^51^.

Control subjects were seen by a neurologist to confirm absence of parkinsonism or tremor. Other screening measures included a medical history, neurological examination, and orthostatic vital signs.

We then explored whether stride-to-stride gait parameters in male subjects in the same age group with clinically established PD and postural instability (n = 5; modified Hoehn and Yahr score of 2.5 or higher and/or self reported gait difficulties; H&Y 2.6 + −0.7; age 65.6 +/− 14.2; height 178.8 +/− 8.9 cm; weight 90.3.6 +/− 4.7 kg; MoCA 24.8 +/− 1.9, range 22–27) deviated from male control data (n = 9, as above).

Subjects were instructed to walk at preferred speed (2 trials), and at slow, very slow, fast, and very fast speeds (1 trial each). In contrast to mice, human subjects maintain relatively steady speeds within and among trials, leading to clustering of data points for each trial, but are able to modulate trial speed on command. After scoring of the trials, we extracted or calculated stride-to-stride gait parameters of the right leg, and visualized data similar to the mouse datasets. We did not apply formal comparative statistical analysis to these datasets.

### Data availability statement

The data that support the findings of this study are available from the corresponding author upon reasonable request.

### Code Availability

Custom Matlab code used for scoring of mouse gait collected on a custom made runway is available from the corresponding author upon reasonable request.

## Electronic supplementary material

Supplementary Figures S1–S3, Supplementary Tables S1–S3
