# Supplementary material for: A translational approach to capture gait signatures of neurological disorders in mice and humans
Source: Sci Rep. 2017 Jun 12;7:3225. doi: 10.1038/s41598-017-03336-1 (PMC5468293; doi:10.1038/s41598-017-03336-1)
Supplement: Supplementary file 1 — Supplementary Figures S1–S3, Supplementary Tables S1–S3 [file 41598_2017_3336_MOESM1_ESM.pdf]

A translational approach to capture gait signatures of neurological disorders in mice and humans

Lauren Broom[1], Brian A Ellison[1], Audrey Worley[1], Lara Wagenaar[1], Elina Sörberg[1], Christine Ashton[1], David A Bennett[2], Aron S Buchman[2], Clifford B Saper[1], Ludy C Shih [1], Jeffrey M Hausdorff [3], Veronique G VanderHorst* [1]

[1] Department of Neurology, Division of Movement Disorders, Beth Israel Deaconess Medical Center, Harvard Medical School, Boston, MA 02215, USA

[2] Rush Alzheimer's Disease Center, Rush University Medical Center, Chicago, Il 60612, USA

[3] Center for the Study of Movement Cognition and Mobility, Tel-Aviv Sourasky Medical Center, Tel Aviv 64239, and Sagol School of Neuroscience and Sackler Faculty of Medicine, Tel Aviv University, Tel Aviv, Israel

Corresponding author: Veronique VanderHorst

Beth Israel Deaconess Medical Center

Center for Life Sciences, CLS 706

Boston MA 02115

Phone: 617 735 3201

Fax: 617 735 3252

Email: vvanderh@bidmc.harvard.edu

Figure S1

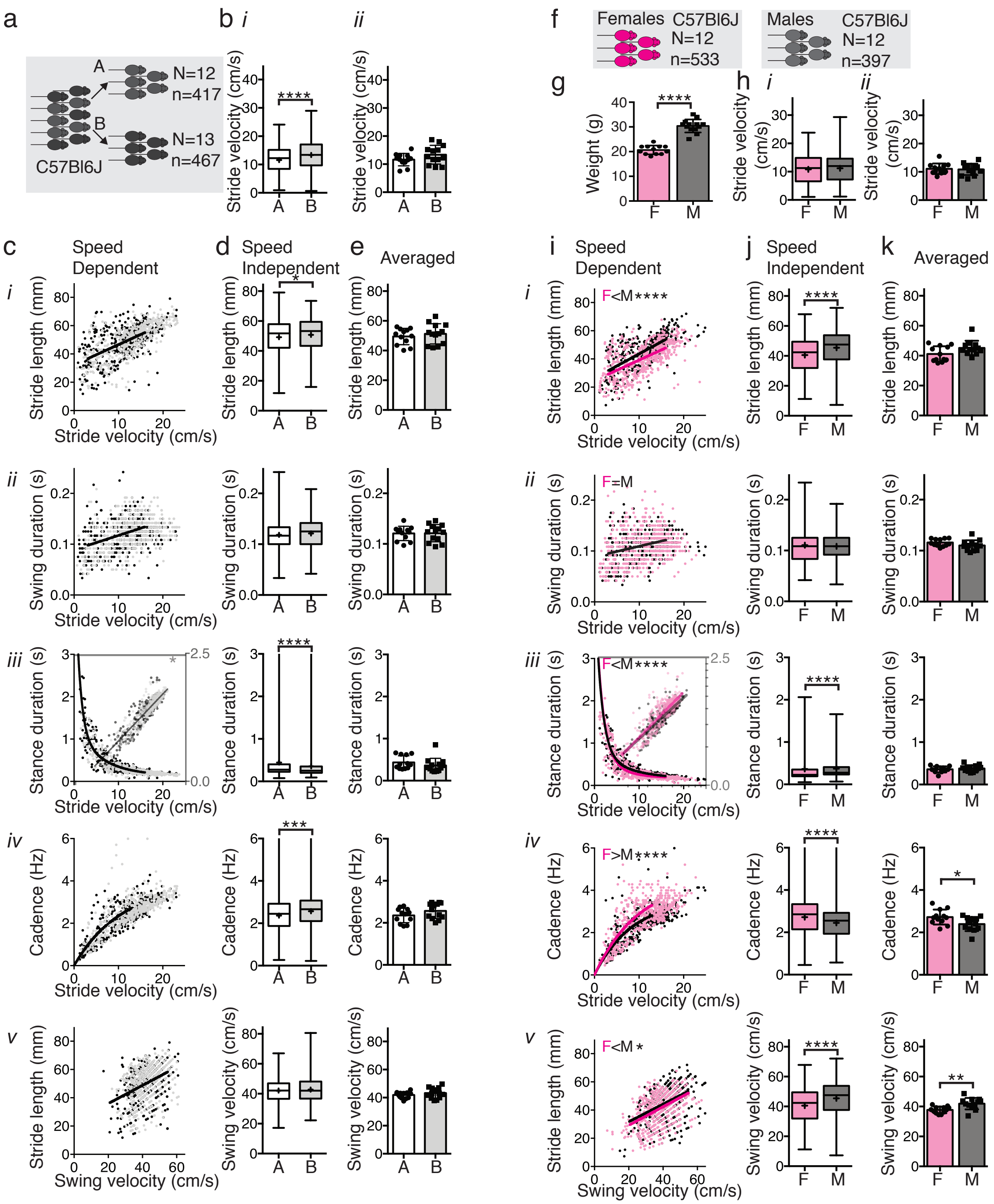

Supplementary Figure S1: Gait parameters during walking: effects of sex, strain and experimental variables
a: Gait parameters were measured in two cohorts with the same genetic background, shipment, age, and sex. **b:** Box and whisker plots (i) show the distribution of stride-to-stride velocity (Mann Whitney Rank test) and (ii) bar graphs with scatter plots represent average stride velocity (two-tailed t-test), with error bars indicating standard deviation. **c:** Relationships of stride length (i), swing duration (ii), stance duration (iii), and cadence (iv) as a function of stride velocity and stride length as a function of swing speed (v) were captured with non-linear regression models using parameters listed in Supplementary Table S2. The fit of one curve to both data sets was compared with the fit of individual curves fit to each dataset (Supplementary Table S3). **d** (i-v) **and e** (i-v)**:** Stride-to-stride data represented as whisker plots (Mann Whitney Rank test, Supplementary Table S3; bars indicate maximum and minimum) and bar graphs with scatter plots represent averaged data (unpaired 2-tailed t-test, Supplementary Table S3; bars indicate standard deviation). **f:** Gait parameters were measured in two cohorts with the same genetic background and age, but different sex. **g:** Bar graphs with scatter plots showing the average weight in the female and male cohorts (2 tailed unpaired t-test; Supplementary Table S3). **h** (i-ii), **I–k** (i-v): legends as in b-e, Supplementary Table S3.

Figure S2

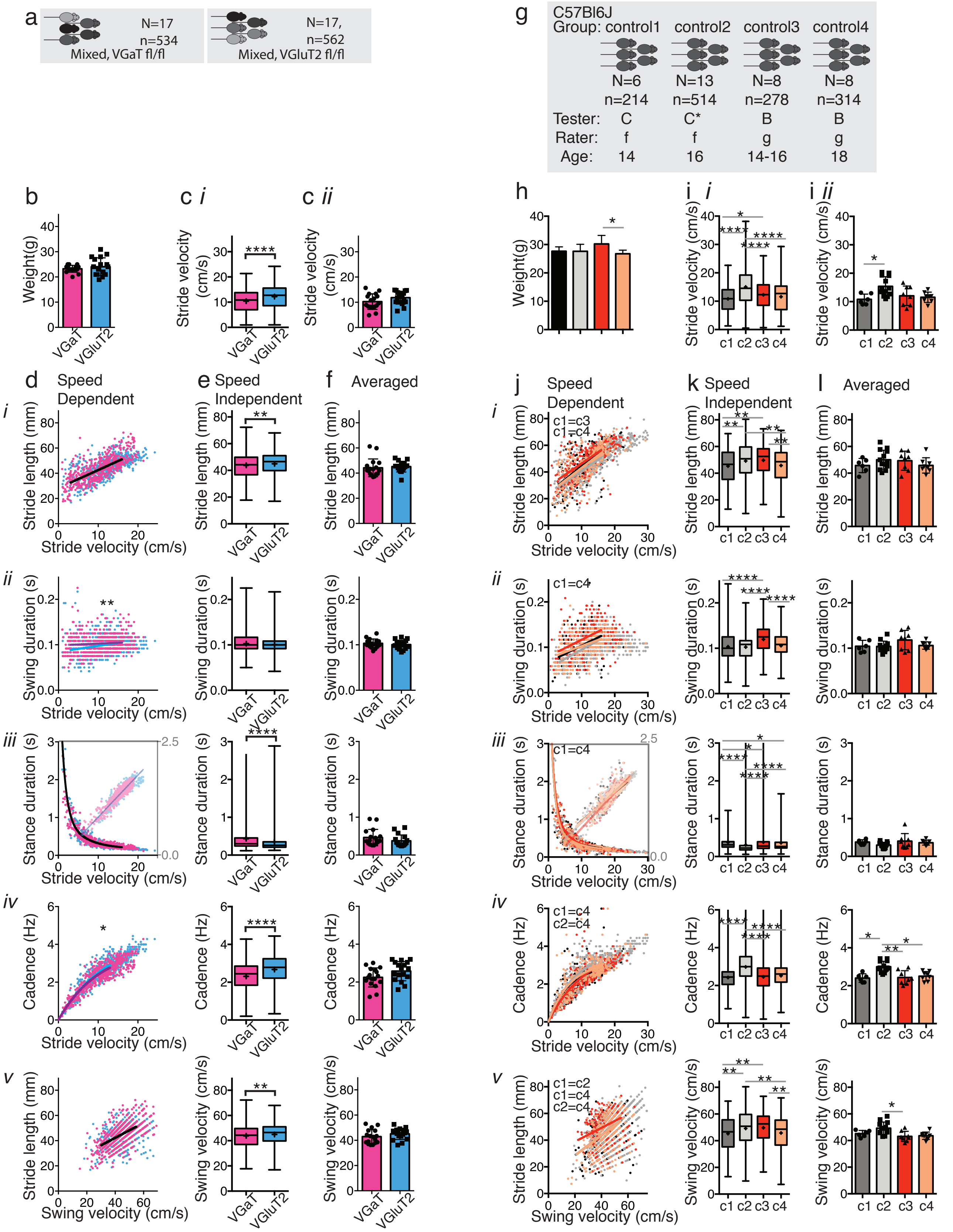

a
N=17
n=534
Mixed, VGaT fl/fl
N=17,
n=562
Mixed, VGluT2 fl/fl
g
C57Bl6J
Group: control1 control2 control3 control4
N=6 N=13 N=8 N=8
n=214 n=514 n=278 n=314
Tester: C C* B B
Rater: f f g g
Age: 14 16 14-16 18
b
Weight(g)
VGaT
VGluT2
c i
c ii
Stride velocity (cm/s)
h
i i
i ii
c1 c2 c3 c4
d Speed Dependent
e Speed Independent
f Averaged
j Speed Dependent
k Speed Independent
l Averaged
Stride length (mm)
Stride velocity (cm/s)
c1=c3
c1=c4
Swing duration (s)
Stance duration (s)
Cadence (Hz)
c2=c4
Swing velocity (cm/s)
c1=c2

Supplementary Figure S2: Gait parameters during walking: effects of strain and experimental variables
a: Gait parameters were measured in cohorts of male mice with different mixed genetic backgrounds, with otherwise similar experimental conditions. **b:** Weight comparisons between both groups depicted in bar graphs with scatter plots (two-tailed t-test; error bars indicate standard deviation). **c:** Box and whisker plots (i) show the distribution of stride-to-stride velocity (Mann Whitney Rank test; Supplementary Table S3) and (ii) bar graphs with scatter plots represent average stride velocity (2-tailed t-test; Supplementary Table S3), with error bars indicating standard deviation. **d:** Relationships of stride length (i), swing duration (ii), stance duration (iii), cadence (iv) as a function of stride velocity and stride length as a function of swing speed (v) were captured with non-linear regression models using parameters listed in Supplementary Table S2. The fit of one curve to both data sets was compared with the fit of individual curves fit to each dataset (Supplementary Table S3). **e and f** (i-v)**:** Stride-to-stride data represented as whisker plots (Mann Whitney Rank test; Supplementary Table S3) and bar graphs with scatter plots represent averaged data (unpaired 2-tailed t-test; Supplementary Table S3).
g: Gait parameters were measured in 4 cohorts of male mice with the same genetic background and age range, but each from a different shipment and with partially different testers and raters. In contrast to all other datasets, a male assistant was present during gait testing of cohort 2. **h:** Comparison of weight among groups depicted in bar graphs with scatter plots (ANOVA, followed by Dunn's post hoc test; error bars indicate standard deviation; Supplementary Table S3). **i:** Box and whisker plots show the distribution of stride-to-stride velocity (Kruskall Wallis, Supplementary Table S3; bars indicate maximum and minimum) and bar graphs with scatter plots representing average stride velocity (ANOVA, followed by Dunn's post hoc test, Supplementary Table S3; error bars indicate standard deviation). **j:** Relationships of stride length (i), swing duration (ii), stance duration (iii), cadence (iv) and swing speed (v) as a function of stride velocity. The fit of one curve to both data sets was compared with the fit of individual curves fit to each dataset (Supplementary Table S3). **k** (i-v)**:** The distribution of the stride-to-stride data among cohorts in box and whisker plots (Kruskall Wallis, followed by Dunn's multiple comparison test; Supplementary Table S3). **l** (i-v)**:** The distribution of averaged data among cohorts in bar graphs with scatter plots (ANOVA, followed by post hoc Dunn's multiple comparison test; Supplementary Table S3).

Figure S3

a Example I, straight line fit:

Samples: Stride length and stride velocity of datasets 4 and 5 (Supplementary Table S1)
Fitting Parameters for Stride length, Supplementary Table S2:
Straight line, No Constraint , Range 3 to 16 cm/s (Supplementary Table S2)
i. Organize data: XY table: Enter and plot a single Y for each X point
Column A: stride length dataset 4 with associated stride velocity in Column X
Column B: stride length dataset 5 with associated stride velocity in Column X
ii. Analyze: Choose type and datasets:
Non-linear regression of data sets in columns A and B
iii. Fit: Choose Equation and fitting method:
Straight line; least squares fit
iv. Compare: Does one curve adequately fit all the data sets?
Extra sum of squares F test and set P value
v. Constrain: Yintercept no constraint, Slope no constraint
vi. Range: do not fit points when X is less than 3
do not fit points when X is greater than 16
vii. Fit the curve Result: One curve for all datasets

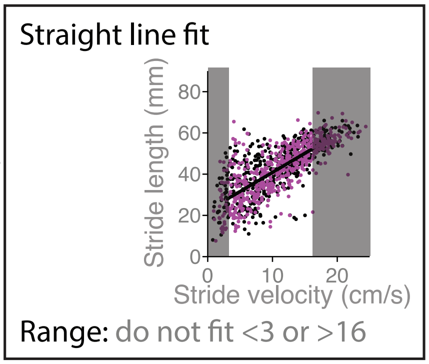

b Example II, one phase or two phase associaton fit:

Samples: Cadence and stride velocity of datasets 16 and 17 (Supplementary Table S1)
Fitting Parameters for cadence, Supplementary Table S2:
One phase association, Constraint Y0=0, Range 0 to 13 cm/s
i. Organize data: XY table: Enter and plot a single Y for each X point
Column A: cadence dataset 16 with associated stride velocity in column X
Column B: cadence dataset 17 with associated stride velocity in column X
ii. Analyze: Choose type and datasets:
Non-linear regression of data sets in columns A and B
iii. Fit: Choose Equation and fitting method:
Equation: 1 phase association; least squares fit
iv. Compare: Does one curve adequately fit all the data sets?
Extra sum of squares F test and set P value
v. Constrain: Y0=0, Plateau no contraint
vi. Range: do not fit points when X is less than 0
do not fit points when X is greater than 13
vii. Fit the curve Result: Different curve for each dataset

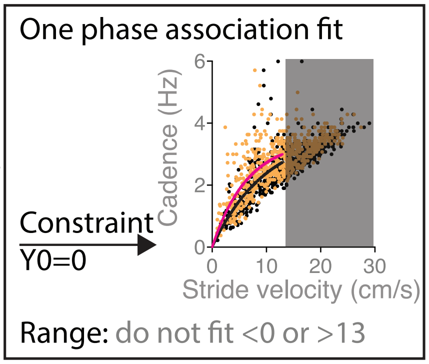

Supplementary Figure 3: Step-by-step guide for the application of curve fitting parameters to compare two datasets

a: Example I summarizes the sequential steps (i-vii) necessary to compare 2 datasets that fit a straight line, i.e. stride length, using Graphpad Prism software. Samples are taken from datasets 4 and 5 (Supplementary Table S1; see also Figure 2). Fitting parameters relevant for this example (indicated in red) as well as for other datasets that match straight lines are summarized in Supplementary Table S2. **b:** Example II summarizes the sequential steps (i-vii) necessary to compare 2 datasets that fit a one phase association, i.e. cadence. Samples are taken from datasets 16 and 17 (Supplementary Table S1; see also Figure 3). Fitting parameters relevant for this example (indicated in red) as well as for datasets that fit two phase associations, i.e. stance duration, are summarized in Supplementary Table S2.

Supplementary Table 1: Overview of mouse datasets

Dataset	Genotype	Sex	#/cohort	Age (wks)	Equipment	Tester	Rater	Condition/Purpose
**1**	C57Bl6/J;129P3/J	M	18	10-12	Runway A	A	d	Large versus Small runway
**2**	C57Bl6/J;129P3/J	M	16	14-18	Runway B	A	a	Large versus Small runway
**3**	C57Bl6J	M	14	10	Runway A	B	e	Large versus Small runway
**4**	C57Bl6J	M	15	10	Runway A	B	e	Within group reproducibility
**5**				11	Runway A	B	e	Within group reproducibility
**6**	C57Bl6J	M	12	14-16	Runway A	B	e	Sex comparison; Reproducibility
**7**	C57Bl6J	F	12	15	Runway A	B	e	Sex comparison
**8**	VGaT$^{fl/fl}$, C57Bl6/J;129P3/J	M	17	10-12	Runway A	A	d	Genetic background; Reproducibility
**9**	VGluT2$^{fl/fl}$, C57Bl6/J;129P3/J	M	17	10-12	Runway A	A	d	Genetic background; Reproducibility
**10**	C57Bl6J	M	6	14	Runway A	C	f	Baseline; Reproducibility
**11**				17	Runway A	C	f	Disease model: 6-OHDA
**12**	C57Bl6J	M	13	16	Runway A	C*	f	Baseline; Reproducibility
**13**				20	Runway A	C*	f	Disease model: 6-OHDA
**14**	C57Bl6J	M	12	14-18	Runway A	B	g	Baseline; Sex comparison; Reproducibility
**15**				17-22	Runway A	B	g	Disease model: 6-OHDA
**16**	C57Bl6J	M	17	14-16	Runway A	B	g, e	Baseline; Reproducibility
**17**				24	Runway A	B	g, e	Disease model: s.c. MPTP
**18**	C57Bl6J	M	10	10	Runway A	C	f	Baseline
**19**				28	Runway A	C	f	Disease model: AAV α-synuclein mPMRF
**20**				28	Runway A	C	f	Control: AAV-GFP mPMRF

* Male assistant was present during testing. All testers were female.

Supplementary Table 2:

Regression analysis parameters for gait measures in mice

**Gait parameter**	**Curve**	**Constraints**	**Range**
Stride length<br>Y: Stride length (mm)<br>X: Stride velocity (cm/sec)	Straight line	No constraints	3 to 16 cm/s
Swing duration<br>Y: Swing time (sec)<br>X: Stride velocity (cm/sec)	Straight line	No constraints	3 to 16 cm/s
Stance duration<br>Y: Stance time (sec)<br>X: Stride velocity (cm/sec)	Two phase association	Plateau 0<x<0.17<br>Y0=6, Males<br>Y0=5, Females	0- 3 to 16 cm/s
Cadence (Step frequency)<br>Y: Cadence (Hz)<br>X: Stride velocity (cm/sec)	One phase association	Y=0	0 to 13 cm/s
Stride length as a function of Swing speed (Stride length /Swing time)<br>Y: Stride length (mm)<br>X: Swing velocity (cm/sec)	Straight line	No constraint	20 to 55 cm/s
Log stance duration<br>Y: 0.9-log (stance)<br>X: (log speed)+0.6	Straight line	No constraint	Full

Table 3 Supplemental
Statistical results

**Figure 1 C**	Runway effect					
Test	Mann-Whitney test		unpaired 2-tailed t-test		F test	
	U	p	t(df)=	p	F (DFn, DFd)	p
Stride velocity	10290	< 0.0001	t=7.479 df=32	< 0.0001	NA	NA
Stride length	9560	< 0.0001	t=7.750 df=32	< 0.0001	NA	NA
Swing duration	30706	< 0.0001	t=3.888 df=32	0.0005	NA	NA
Stance duration	12855	< 0.0001	t=5.558 df=32	< 0.0001	NA	NA
Cadence	16577	< 0.0001	t=6.492 df=32	< 0.0001	NA	NA
Swing speed	9633	< 0.0001	t=4.956 df=32	< 0.0001	NA	NA
log stance	12639	< 0.0001				

**Figure 2 C-G**	Reproducibility within group					
Test	Mann-Whitney test		paired 2-tailed t-test		F test	
	U	p	t(df)	p	F (DFn, DFd)	p
Stride velocity	144638	0.43	t=0.5938 df=13	0.56		
Stride length	147342	0.79	t=0.1460 df=13	0.89	1.059 (2,812)	0.35
Swing duration	144149	0.32	t=0.3352 df=13	0.74	0.6404 (2,813)	0.53
Stance duration	141390	0.12	t=0.8082 df=13	0.43	1.949 (4,811)	0.10
Cadence	142406	0.22	t=0.8312 df=13	0.42	1.157 (2,665)	0.31
Swing speed	148331	0.81	t=1.810 df=13	0.09	0.6214 (2,1058)	0.54
log stance					4.446 (2,1090)	0.01

**Figure 3**	MPTP					
Test	Mann-Whitney test		paired 2-tailed t-test		F test	
	U	p	t(df)	p	F (DFn, DFd)	p
Stride velocity	178633	0.003	t=0.7110 df=16	0.49	-	-
Stride length	139905	< 0.0001	t=2.771 df=16	0.01	40.80 (2,867)	< 0.0001
Swing duration	145920	< 0.0001	t=2.918 df=16	0.01	17.23 (2,867)	< 0.0001
Stance duration	189027	0.21	t=0.03451 df=16	0.97	18.72 (4,908)	< 0.0001
Cadence	171374	< 0.0001	t=1.254 df=16	0.23	19.18 (2,638)	< 0.0001
Swing speed	139905	< 0.0001	t=1.503 df=16	0.15	48.52 (2,1150)	< 0.0001
log stance	-	-	-	-	48.20 (2,1252)	< 0.0001

**Open Field**	paired, 2 tailed t test	
	t(df)	p
distance	t=1.528 df=16	0.15
time immobile	t=1.164 df=16	0.26
average speed	t=1.098 df=16	0.29

**Figure 4**	6OHDA					
Test	Mann-Whitney test		paired 2-tailed t-test		F test	
	U	p	t(df)	p	F (DFn, DFd)	p
Stride velocity	493584	< 0.0001	t=1.340 df=28	0.19	-	-
Stride length	450773	< 0.0001	t=2.975 df=28	0.006	13.92 (2,1545)	< 0.0001
Swing duration	526538	0.08	t=0.05437 df=28	0.96	1.346 (2,1541)	0.26
Stance duration	543950	0.62	t=0.3241 df=28	0.75	3.934 (4,1540)	0.004
Cadence	550744	0.87	t=0.5386 df=28	0.59	2.361 (2,1187)	0.09
Swing speed	450571	< 0.0001	t=2.731 df=28	0.01	12.56 (2,1768)	< 0.0001
log stance	-	-	-	-	27.35 (2,2108)	< 0.0001
			2 post animals no gait due to turning			

**Open Field**	paired, 2 tailed t test	
	t(df)	p
distance	t=2.787 df=30	0.009
time immobile	t=2.686 df=30	0.01
average speed	t=4.411 df=30	0.0001

**Figure 5**							
Test	Kruskal-Wallis		Dunn's			Kruskal-Wallis	
	H	p	Base vs GFP	Base vs asyn	GFP vs asyn	KW	p
Stride velocity	13.4	0.001	*	**	ns	2.086	0.37
Stride length	18.2	0.0001	ns	****	*	0.8233	0.69
Swing duration	16.1	0.0003	ns	**	**	1.429	0.51
Stance duration	16.4	0.0003	***	*	ns	0.4519	0.81
Cadence	13.8	0.001	***	ns	ns	1.96	0.40
Swing speed	18.2	0.0001	ns	***	*	0.8363	0.38

Family p 0.05	Base versus GFP		Base versus asyn		GFP vs asyn	
Correction 0.01	F test		F test		F test	
	F (DFn, DFd)	p	F (DFn, DFd)	p	F (DFn, DFd)	p
Stride length	4.497 (2,421)	0.01	4.454 (2,485)	0.01	8.035 (2,350)	0.0004
Swing duration	1.547 (2,419)	0.21	4.753 (2,485)	0.009	5.850 (2,346)	0.003
Stance duration	1.345 (4,467)	0.25	7.686 (4,541)	< 0.0001	2.902 (4,346)	0.02
Cadence	1.297 (2,371)	0.27	3.659 (2,439)	0.03	5.284 (2,312)	0.006
Swing speed	0.1101 (2, 514)	0.90	6.481 (2, 578)	0.002	5.591 (2,368)	0.004
log stance	3.531 (2,616)	0.03	6.163 (2,708)	0.002	4.834 (2,440)	0.008

**Figure 6**					
Test	Kruskal-Wallis		Dunn's		
	H	p	Ctrl A-CtrlB	Ctrl B-PD B	Ctrl A-PD B
Stride velocity	180	< 0.0001	< 0.0001	< 0.0001	< 0.0001
Stride length	326	< 0.0001	0.16	< 0.0001	< 0.0001
Swing duration	84	< 0.0001	< 0.0001	> 0.9999	< 0.0001
Stance duration	260	< 0.0001	< 0.0001	0.009	< 0.0001
Cadence	331	< 0.0001	< 0.0001	< 0.0001	< 0.0001
Swing speed	326	< 0.0001	0.14	< 0.0001	< 0.0001

**Figure 1S A-E**	Reproducibility using litter mates					
Test	Mann-Whitney test		unpaired 2-tailed t-test		F test	
	U	p	t(df)	p	F (DFn, DFd)	p
Stride velocity	79150	< 0.0001	t=1.552 df=23	0.13		
Stride length	89172	0.03	t=0.7256 df=23	0.48	2.346 (2,616)	0.10
Swing duration	91977	0.17	t=0.08439 df=23	0.93	1.002 (2,615)	0.37

Stance duration	80440	< 0.0001	t=1.109 df=23	0.28	0.9308 (4,658)	0.45
Cadence	83266	0.0002	t=1.547 df=23	0.14	0.6179 (2,443)	0.54
Swing speed	95327	0.59	t=1.056 df=23	0.30	2.374 (2,818)	0.09
log stance					1.248 (2,880)	0.29

**Figure S 1F-K**	Males versus females, speed similar					
Test	Mann-Whitney test		2-tailed t-test		F test	
	U	p	t(df)	p	F (DFn, DFd)	p
Stride velocity	112347	0.41	t=0.6088 df=22	0.55	-	-
Stride length	79457	< 0.0001	t=1.978 df=22	0.06	23.27 (2,741)	< 0.0001
Swing duration	102528	0.54	t=1.620 df=22	0.12	2.271 (2,739)	0.10
Stance duration	79002	< 0.0001	t=0.6749 df=22	0.51	7.601 (4,736)	< 0.0001
Cadence	84819	< 0.0001	t=2.359 df=22	0.03	12.86 (2,551)	< 0.0001
Swing speed	79457	< 0.0001	t=3.234 df=22	0.004	3.193 (2,870)	0.04
log stance	-	-	-	-	40.72 (2,931)	< 0.0001

**Figure 2S A-F**	mixed genetic background					
Test	Mann-Whitney test		2-tailed t-test		F test	
	U	p	t(df)	p	F (DFn, DFd)	p
Stride velocity	117398	< 0.0001	t=1.613 df=32	0.12	-	-
Stride length	133705	0.002	t=0.4683 df=32	0.64	2.522 (2,858)	0.08
Swing duration	140569	0.07	t=0.7976 df=32	0.43	6.885 (2,858)	0.001
Stance duration	112320	< 0.0001	t=1.357 df=32	0.18	1.622 (4,854)	0.17
Cadence	108807	< 0.0001	t=1.910 df=32	0.07	4.474 (2,665)	0.01
Swing speed	138131	0.002	t=1.276 df=32	0.21	1.766 (2,875)	0.17
log stance	-	-	-	-	0.3305 (2,1092)	0.72

**Figure 2S G-L**	4 unmatched baseline groups					
Test	Kruskall Wallis	4 groups		Kruskall Wallis		
	KW	p	Dunn's	KW	p	Dunn's
Stride velocity	126.9	< 0.0001	see Fig 2S i-i	10.21	0.02	see Fig 2S i-ii
Stride length	27.3	< 0.0001		2.666	0.45	
Swing duration	60.1	< 0.0001		2.333	0.51	
Stance duration	126.9	< 0.0001		4.691	0.20	
Cadence	142.3	< 0.0001		17.02	0.0007	
Swing speed	27.3	< 0.0001		11.36	0.010	
log stance	-	-		-	-	

4 groups	F test	(family 0.05)	<0.001									
	F (DFn, DFd)	p	F (DFn, DFd)	p	F (DFn, DFd)	p	F (DFn, DFd)	p	F (DFn, DFd)	p	F (DFn, DFd)	p
	1 versus 2	1 versus 2	1 versus 3	1 versus 3	1 versus 4	1 versus 4	2 versus 3	2 versus 3	2 versus 4	2 versus 4	3 versus 4	3 versus 4
Stride velocity	-	-										
Stride length	8.847 (2,483)	0.0002	2.700 (2,382)	0.07	1.269 (2,428)	0.28	21.81 (2,505)	< 0.0001	4.937 (2,551)	0.008	7.682 (2,450)	0.0005
Swing duration	5.634 (2,483)	0.004	8.122 (2,382)	0.0004	0.9030 (2,428)	0.41	33.26 (2,505)	< 0.0001	7.490 (2,551)	0.0006	14.95 (2,450)	< 0.0001
Stance duration	1.746 (4,479)	0.14	3.747 (4,378)	0.005	0.2507 (4,424)	0.91	5.842 (4,501)	0.0001	0.9410 (4,547)	0.44	5.184 (4,446)	0.0004
Cadence	5.097 (2,356)	0.007	1.439 (2,382)	0.24	0.4986 (2,428)	0.61	7.769 (2,362)	0.0005	0.5553 (2,376)	0.57	5.605 (2,469)	0.004
Swing speed	0.02371 (2,570)	0.98	12.10 (2,431)	< 0.0001	0.003314 (2,465)	1.00	16.03 (2,649)	< 0.0001	0.06812 (2,683)	0.93	16.06 (2,544)	< 0.0001
